# CRISPR Treatments for AI-Designed Synthetic Viruses: Rapid Programmable Countermeasures for Emerging and Engineered Viruses

**DOI:** 10.3390/v17121588

**Published:** 2025-12-05

**Authors:** Douglas P. Gladue, Alison O’Mahony

**Affiliations:** Seek Labs, 350 W 800 N Suite 220, Salt Lake City, UT 84103, USA

**Keywords:** CRISPR, Cas13, antivirals, synthetic biology, AI, DNA vectors, Cas9, biosecurity, biothreat

## Abstract

The convergence of artificial intelligence and synthetic biology is innovating and accelerating the design of novel viral genomes, expanding both therapeutic opportunities and dual-use risk. This review articulates a countermeasure strategy for emerging and engineered viruses leveraging the programmable CRISPR modality. Building on mounting in vitro and in vivo evidence that Cas9 degrades DNA viruses (e.g., Orthopoxviruses, HSV-1, ASFV), while Cas13 targets RNA viral genomes (e.g., Influenza A, Dengue, RSV), both leading to reduced viremia, diminished disease burden, and alleviated symptoms. Here, we outline a rapid-response pipeline to position CRISPR-based countermeasures in translational and pandemic-response frameworks, linking real-time sequencing to AI-assisted gRNA selection and multiplexed cassette design to achieve viral targeting efficacy. To minimize resistance and off-target risk, we emphasize multi-gRNA cocktails, continuous genomic surveillance, and adaptive gRNA rotation. We also propose governance mechanisms, such as pre-cleared gRNA repositories, transparent design logs, standardized off-target/safety screening, and alignment with evolving nucleic-acid-synthesis screening frameworks to enable emergency deployment while preserving security. Furthermore, compressing the time from sequence to treatment and complementary to vaccines and small-molecule antivirals, CRISPR represents a technologically agile and strategically essential capability to combat both natural outbreaks and AI-enabled biothreats. Collectively, programmable CRISPR antivirals represent an auditable, rapidly adaptable foundation for next-generation biodefense preparedness.

## 1. Introduction

The accelerating convergence of artificial intelligence and synthetic biology (synbio) has expanded the capacity to design, generate, and potentially deploy novel viruses at unprecedented speed. Advances in these technologies offer substantial societal benefits but also carry inherent risks of misuse or unintended harm. For instance, Stanford researchers recently reported using AI models to engineer novel bacteriophage variants from existing lab strains. These variants incorporate safeguards to ensure specificity for bacteria (without harming humans, animals, or plants), serving as an antibiotic alternative and potentially saving lives in cases of drug-resistant bacterial infections. On the risk side, such advances could create viruses that bypass existing biosafety protocols, increasing the potential for bioterrorism or accidental pandemics. For instance, a recent Microsoft study demonstrated an AI approach to generate novel genetic sequences encoding harmful proteins, such as cell death inducers [1]. Of concern was the finding that even with software patches, about 3% of biothreat designs still evaded the screening software used by biotech DNA synthesis suppliers [1]. The potential dual-use capabilities heighten the urgent and critical need for equally adaptable and accelerated countermeasures.

This review proposes a rapid-response pipeline from AI-gRNA design to emergency deployment and outlines governance mechanisms, including pre-cleared gRNA repositories for new and emerging priority pathogens, along with streamlined AI-predicted emergency-use gRNAs for optimal off-target and safety potential. Integrating these elements provides an actionable path towards delivering safe, effective, and scalable programmable antivirals suitable for rapid outbreak response.

## 2. AI in Synthetic Virology and the Expanding Threat Landscape

Large-context genome language models have now generated viral genomes with coherent coding architectures and viable phenotypes, including host tropism and replication competence in bacteria [2]. While AI applications have thus far focused on bacteriophages, they mark a qualitative shift where generative models can design viral genomes beyond simple motif shuffling and reconstruction of particles, toward functional novelty and even new organisms. Although this capability expands the potential for new viral designs to help with therapeutic applications, it also opens the door to the development of novel biothreats. While AI lowers the design barrier, significant wet-lab constraints (assembly, rescue, and biosafety) still exist and would require more than computational and technical expertise to design a new lifeform or bioweapon that poses a threat to humans. Nonetheless, this development represents a future reality that may not be that far off and compels the reassessment of technical countermeasures that can be rapidly deployed. In the case of an emergency outbreak or pandemic situation, re-programming CRISPR antivirals with novel gRNA sequences precisely fills the niche where current antivirals simply do not fit. Historical countermeasures, including vaccines, small molecule antivirals, and monoclonal antibodies, require considerable time, effort, and expense to develop as deployable therapeutics. Moreover, only a small percentage of infectious diseases can be successfully managed with these conventional treatments. In contrast, CRISPR-based modalities represent a programmable, rapid-response approach with several advantages over traditional approaches (Table 1).

Generative AI did not create the potential for synthetic viral biothreats on its own; indeed, there have been several historical milestones that shaped today’s synthetic viral landscape (Table 2). In 2002, the first demonstration of de novo synthesis of poliovirus from oligonucleotides was achieved, although at the time, this involved the complicated, error-prone, and laborious stitching together of small pieces of the viral genome [3]. This was followed only one year later by a whole-genome assembly of bacteriophage φX174 also from synthetic oligos [4]. Since then, other labs have followed similar methods for re-constructing different viruses; however, in all cases, whole genome assembly involved a virus that already existed in various laboratories. In 2018, researchers assembled an extinct relative of a smallpox virus, called horsepox virus, simply from purchased DNA fragments. At the time, the researchers were heavily criticized for publishing a step-by-step procedure that could be used to reconstruct smallpox, which raised global biosecurity risks [5]. In 2005, researchers reconstructed and characterized the 1918 influenza A virus that caused the infamous Spanish Flu pandemic by recovering the sequence from historical tissue samples and using a plasmid-based system for all eight segments of influenza. While this was performed under strict biosecurity measures, including laboratory containment and personal safety considerations, the researchers did re-create what had been an extinct version of influenza [6]. Infectious cDNA clones and reverse-genetics systems have existed for decades and effectively generate a wide range of viruses. Most recently, for SARS-CoV and SARS-CoV-2, they enabled reconstruction and variant engineering for research purposes [7,8]. Although for more difficult segmented dsRNA viruses (e.g., bluetongue), reverse genetics required longer development efforts, it now fully supports robust recovery and bioengineering [9]. Technology has also advanced for large dsDNA viruses—for example, yeast-based synthetic genomics methods have enabled genome-scale manipulations of HSV-1 [10], and with the help of a helper virus, recovery of synthetically developed Vaccinia virus [11] and African swine fever virus [12] now exists. Collectively, these efforts definitively prove that sequence-to-virus pipelines are possible across many virus families and are not just limited to bacteriophages that were recently recreated by AI [2]. Moreover, the time to synthesize novel sequences has accelerated exponentially with desktop DNA synthesizers, giving the potential to develop new infectious clones of viruses rapidly. To address the evolving threat landscape of AI-engineered viruses, CRISPR-based antivirals offer a uniquely programmable and rapid-response solution, further discussed below.

## 3. CRISPR Antivirals: A Programmable Firewall Against Synthetic Biology Threats

Unlike small molecules, antivirals, or therapeutic monoclonal antibodies that require a deep understanding of protein function, target selectivity, and biological context-dependent mechanism of action (MoA), CRISPR activity in any mammalian cell relies only on the presence of a short strand of RNA complementary to a short sequence of the target virus genome. This direct MoA enables sequence-specific, multiplexed targeting of viral genes, disrupting replication and lowering disease burden with the unique ability to be rapidly reprogrammed to target and cleave a different virus simply with new gRNAs. CRISPR/Cas13 effectors can degrade RNA viral genomes and transcripts across diverse viruses, including human infectious pathogens and zoonotic strains (e.g., influenza A, coronaviruses, dengue) [13,14,15,16,17,18]. We have recently shown that DNA-targeting Cas9-based multiplexed CRISPR can ablate African swine fever virus to facilitate survival of pigs infected with an otherwise lethal pathogen [19], highlighting the potential to treat other large DNA viruses such as Smallpox and Mpox.

Applications of single-gRNA CRISPR antiviral modalities could create selective pressure that could allow for the emergence of escape variants over time. Consequently, multi-gRNA cassettes designed against highly conserved, functionally constrained target regions raise the mutational hurdle and reduce the likelihood of pressure-based escape variants. For RNA viruses, simultaneous targeting and cleavage at multiple conserved loci often imposes severe fitness costs compared to point mutations that can overcome conventional drugs with a single molecule MoA. Although AI-designed viruses could distribute synonymous changes to avoid CRISPR targeting gRNAs, they cannot circumvent newly designed gRNAs, a capability by which CRISPR systems can keep pace with virus evolution or AI-designed viruses—even in the face of continuously changing biothreats—and represents a unique opportunity to design and deploy a treatment at the speed of an infection.

To prioritize stability and durability, replication-competent DNA-based CRISPR antivirals represent a compelling option, as DNA offers lower cost and greater reliability over RNA platforms, while also allowing for both the Cas and multiplex gRNAs to be delivered as a single payload. DNA-based systems also have the potential for lyophilization and stockpile treatments over the cold-chain that is required for RNA-based delivery systems. However, there is no single delivery mechanism that will fit all biothreats. Different viruses have different tissue tropisms and require different therapeutic strategies. We recently demonstrated the effectiveness of a Cas9 therapeutic using Lipid-nanoparticle delivery for African swine fever, where both the virus and therapeutic were able to interact in the blood, likely in circulating macrophage cells that serve as the primary site of replication for ASFV [19]. However, for respiratory diseases, intranasal or intratracheal administration would likely be required to deliver the treatment to infected respiratory tissues. An alternative delivery option could be Adeno-associated viruses (AAV) to achieve multi-week airway expression of Cas and gRNAs. Other viral vectors could be used for potentially larger payloads, such as helper-dependent adenovirus (HD-Ad), and robust acute expression or non-replicating HSV amplicons with large payload capacity could also be used as a delivery system. Regardless, all delivery platforms will have to be matched to the characteristics of the targeted disease, and multi-site gRNA cassettes remain essential. Safety considerations still need to be determined even in the case of rapid response for emergency outbreaks, such as vector biodistribution, vector immunogenicity (AAV/Ad), off-target, and collateral effects (Cas13). All CRISPR treatments will likely favor non-integrating vectors and time-limited promoters or delivery systems [20] (Table 3). Taken together, we will discuss below the feasibility of CRISPR antivirals for rapid response to new or emerging viruses.

## 4. CRISPR-Enabled Rapid-Response Potential

Upon first attribution of symptoms to a novel pathogen, next-generation sequencing (NGS) platforms must be capable of identifying both known and unknown viral sequences, rather than relying solely on similarity to established viral signatures. The recent emergence of AI-generated baculovirus-like genomes illustrates this challenge: several synthetic sequences were not recognized as viral DNA by conventional bioinformatic filters [2]. This underscores the importance of recognizing that previously unseen genomic signatures may represent AI-engineered biothreats. In most outbreaks involving highly pathogenic viruses, large viral burdens within the host enable early detection through clinical symptoms or post-mortem analysis. Once a unique nucleotide signature is identified, CRISPR-based targeting sequences (gRNAs) can be computationally designed within minutes, so long as the appropriate informatics infrastructure is in place.

Advances in de novo DNA synthesis now allow for commercial and benchtop systems to produce short nucleic acid fragments in near-real time. These gRNA sequences can be rapidly assembled into delivery backbones, such as viral vectors or nanoparticle formulations. The urgency and scale of production would naturally be dictated by disease morbidity and mortality. Despite the theoretical feasibility of this “minutes-to-therapeutic” model, several major challenges remain. These include scalable manufacturing infrastructure, continuous genomic surveillance to detect escape mutants, and automated regeneration of gRNA cocktails guided by AI-assisted analysis for both safety and efficacy. Synthetic sequences should be screened under established synthesis-monitoring frameworks [28,29]. Moreover, AI-enabled gRNA design tools must incorporate risk assessments for host off-target effects, predictive modeling for cleavage efficacy, and conserved-region prioritization to ensure robust resistance to viral evolution [30,31].

### Computational Resources for CRISPR gRNA Design

A growing ecosystem of computational resources supports rapid, accurate CRISPR gRNA design across nucleases and host systems. It is useful to distinguish resources that distribute pre-vetted gRNA from those that enable de novo design. PAC-MAN (Prophylactic Antiviral CRISPR in huMAN cells) refers to a Cas13-based antiviral strategy rather than a standalone design algorithm. Following that study, public portals, such as the Stanford CRISPR PAC-MAN site, provided curated, “family-covering” crRNA pools (e.g., for Coronaviridae) intended for immediate experimental use, emphasizing breadth and prior validation rather than custom design [32]. In parallel, the New York Genome Center’s Cas13Design supports custom gRNA selection for Cas13d, allowing users to tailor crRNAs to new or evolving RNA viral genomes [33]. In practice, a rapid pipeline often combines these assets, drawing on curated panels for speed while invoking custom design when coverage gaps or emerging variants demand novel gRNA.

For the development of rapid response pipelines, Seek Labs uses BioSeeker™ and to create a disease atlas of curated genomic targets spanning the majority of clinically relevant infectious viruses, which can accelerate selection of viral targeting gRNAs by pre-mapping a set of gRNAs that pan-target all isolates of a particular virus. Complementing this, BioSeeker generates “safe-gRNA” from multi-host genomic data to minimize off-target risks, providing candidate sets that are both efficacious and deployment-ready in diverse biological contexts.

Beyond these, several widely used platforms provide design, analysis, and workflow integration. Tools such as CHOPCHOP [34], EuPaGDT [35], and TIGER [36] offer web-based gRNA design and annotation across multiple nucleases and organisms, incorporating common scoring and off-target heuristics. Commercial and developer ecosystems (e.g., IDT, Synthego, Benchling) can add capabilities for synthesis-ready formatting and cloning strategy output. New research-driven resources from the Arc Institute [37] and others continue to refine predictive models and benchmarking datasets. Together, these assets span the spectrum from curated, plug-and-play gRNA pools to fully custom, model-driven design, enabling teams to match turnaround time and rigor to the demands of each project.

## 5. Governance and ELSI for AI-Enabled Synthetic Virology: DURC to Digital Biosecurity

Synthetic biology (SynBio) lies at the cutting edge of bioengineering advances, enabling the design and creation of custom genetic systems and novel organisms to address complex challenges, such as targeted therapies and ultra-sensitive biosensors [38]. The integration of AI supercharges these efforts with accelerated sequence predictions, optimized designs, and scaled workflows, paving the way for powerful defensive tools to counter biothreats [39,40]. To mitigate the misuse of SynBio and AI-bioengineering, risk assessments are run to inform the Biological Weapons Convention (BWC) on potential threats [14,19,20,28,41]. Existing oversight from the Dual Use Research of Concern (DURC), Enhanced Pathogens with Pandemic Potential (ePPP/PEPP) policies, and sequence-data governance (e.g., NIH GDS, GDPR, OECD guidance) all emphasize an integrated, holistic approach involving government, academia, and industry [30,41,42,43,44,45,46,47,48,49]. However, when innovation and capabilities expand rapidly—including digital diffusion of models generating novel sequences or when parts of a workflow occur outside traditional oversight structures—risks can outpace effective responses [30,39,40,44,47].

### 5.1. What Current Frameworks Cover—And Where They Fall Short

Oversight agencies such as DURC and ePPP require institutions to assess whether legitimate R&D experiments could be misused to create, enhance, or disseminate dangerous pathogens [42,43,44]. Furthermore, they rely on these same institutions to manage risks through review, mitigation, or prohibition [42,43]. These entities work best when projects are bounded (defined organism, methods, materials) and when risk is confined to physical lab spaces [30,44]. They often fall short when design, optimization, and initial screening are in silico, when cloud-based or third-party providers execute steps, and when the AI models’ access, prompts, or outputs are not explicitly governed or do not have robust stewardship programs in place [30,39,47]. Data access or sharing rules can reduce indiscriminate sharing, but few policies comprehensively address data quality, security, compliance (i.e., data integrity, access/control points, metadata management, prompt filters, audits and monitoring, etc.) [40,43,47], or supply-chain screening for sequence-to-physical translation [45,46,50].

### 5.2. Historical Stress Tests and What They Changed

A series of landmark studies have shaped the Synthetic biology space: 1918 influenza reconstruction [51]; H5N1 airborne transmission in ferrets [52]; and synthetic horsepox from DNA fragments [5]. Together, these studies demonstrated that high scientific value could coexist with substantive risk in allowing an extinct virus to be recreated. More recent AI-enabled sequence design [2], coupled with inexpensive benchtop or ready-to-order DNA synthesis suppliers, has exposed gaps in policies and procedures required to advance research and also minimize risk. Table 4 summarizes representative cases, specific DURC/ePPP concerns, and observable policy outcomes that highlight the critical and urgent need to extend oversight from wet-lab activities to digital design and synthesis capabilities.

### 5.3. Digital Biosecurity: The AI Risk Surface

AI systems can (i) generate novel or optimized sequences with pathogenic traits, (ii) design constructs that slip past naïve sequence filters, (iii) translate literature and programming code into step-by-step protocols, and (iv) distribute capability beyond strictly regulated environments. Without model-level controls, the same tools that accelerate beneficial design can markedly reduce barriers to misuse. This expanding digital risk surface argues for governance that reaches upstream of research lab oversight to AI modeling, data access, prompt and output filtering, and auditability. Additionally, rigorous screening of nucleotide synthesis orders is required for a policy that keeps pace with the AI design of potential biothreats.

## 6. Examples of Existing Viral Pathogens for CRISPR Antivirals

### 6.1. Avian and Pandemic Strains of Influenza

Seasonal Influenza cases in the US over the past 3 years (2022–2025) have shown increasing severity, and the most recent 2024–2025 season was classified by the CDC as “high severity” with the highest estimated disease burden in the past decade based on outpatient visits, hospitalizations, and deaths [57,58]. Globally, the WHO estimates 290,000–650,000 annual respiratory deaths from seasonal influenza, with recent seasons exacerbated by dominant A(H1N1) pdm09 and A(H3N2) strains for which the CDC reported moderate overall effectiveness (VE) at 30–60% [59,60]. Coupled with this is the ongoing global outbreak of the highly pathogenic avian influenza A(H5N1) clade2.3.4.4b, which began in 2020 and has intensified from 2022 onward, affecting wild birds, domestic poultry, livestock, and humans [61]. As of February 2025, it has spread to every continent except Australia [61]. While no sustained mammal-to-mammal or human-to-human transmission has been reported, risk surveillance is high due to mammalian adaptations [62]. Together, these incidents serve as compelling reminders that existing respiratory viral diseases continue to pose a significant threat, where CRISPR modalities could be a potential preparedness treatment option.

CRISPR antivirals offer a rapid design, easily reprogrammable, sequence-directed way to target the influenza genome, disrupt viral replication, and limit cross-species spread [12,14]. By designing multiplexing Cas13 gRNAs to target and cleave conserved RNA genome segments, we can rapidly update gRNAs and reprogram the CRISPR system as genome sequences shift, an approach that not only mediates direct viral ablation but could also be used to complement vaccines and small molecule polymerase/neuraminidase inhibitors [12,14]. CRISPR antivirals remain agnostic to antigenic drift/shift and could function in multiple biological contexts. Translationally, delivery strategies can utilize upper-airway routes already validated for nucleic acid-based modalities: intranasally administered lipid nanoparticles (or inhaled aerosols) that deliver payload to airway epithelium and immune cells, enabling post-exposure treatment or potential ring prophylaxis in farms and healthcare settings [63,64]. The expanding mammalian footprint of Influenza A underscores a need for both commercially available therapeutics along with emergency-use gRNA stockpiles, which would not have evolutionary pressure if not in use. Additionally, CRISPR could decrease the risk from AI-designed viruses that could evade treatment. By using a platform approach, new gRNAs could be finalized within days of a new sequence or AI-designed variant.

Mounting evidence supports this strategy. Freije et al. (CARVER) demonstrated robust suppression of influenza A replication using human cell-based in vitro models and the CRISPR/Cas13b system with multiplexed gRNAs to target the conserved NP (nucleoprotein) and M (matrix) viral genes [21]. This study was independently validated using the PAC-MAN framework to take a more prophylactic CRISPR deployment in human lung epithelial cells, targeting conserved IAV gene regions, particularly in H1N1, with an eye toward broader, pan-influenza strategies [32]. Alternatively, we used our proprietary AI platform, Bioseeker, using a multiplex approach where all gRNAs achieve pan-targeting CRISPR for influenza A with predicted high efficacy. Translationally, mRNA-encoded Cas13a with PB1/PB2-targeting gRNAs, delivered to mice via nebulization post-infection, was shown to reduce influenza A gene expression consistent with therapeutic application [13]. Additional studies report site-specific genomic RNA degradation of IAV using mRNA-Cas13a and continued efficacy signals, supporting an intranasal or inhaled LNP/polymer administration as a viable route for treating acute flu [15]. Newer preclinical work also suggests cell-selective or targeted LNP (tLNP) delivery formulations of Cas13d can achieve large reductions in viral load, leading to complete survival in otherwise lethal mouse models, highlighting the potential for airway-targeted formulations that improve potency and safety as a selective antiviral treatment [16]. Taken together, these results support the promise of a response-ready, multiplexed Cas13 therapeutic for the current H5N1 panzootic [13] that could also be adapted for newly emerging strains or AI-designed biothreats.

### 6.2. Smallpox and Monkeypox

With recent outbreaks of mpox and the ongoing biothreat potential of smallpox, a CRISPR-Cas9 therapeutic for orthopoxviruses could be used to target conserved viral DNA using multiplexed gRNAs to inhibit replication, which could complement tecovirimat, which now shows both on-therapy and transmitted resistance [65,66]. Proof-of-concept with vaccinia (a surrogate for other orthopoxviruses) demonstrated that Cas9 gRNAs against conserved regions markedly reduced viral titers in human cells, including when delivered by AAV vectors [67]. For deployment, nonviral formulations enable stockpile-ready manufacturing and lesion-proximal administration: lipid nanoparticles or dissolvable microneedle patches can localize editors to skin and mucosa, offering intradermal/topical options for mpox lesions and systemic options for severe disease [68]. Together, Cas9 with multiplexed, pre-vetted gRNA sequences against conserved orthopoxviruses targets provides a programmable antiviral that can be retargeted within days as new mpox lineages, including potential tecovirimat resistance [66,69], emerge or potential AI-engineered variants that circumvent current countermeasures to orthopoxviruses. While CRISPR systems have been used experimentally to target both RNA and DNA viral genomes, the limitations for the broad use of CRISPR antivirals will be covered in the next section.

## 7. Current Limitations for CRISPR Antivirals

### 7.1. Targeted Tissue Delivery

Current CRISPR delivery methods for treatment strategies largely rely on two systems with very different risk profiles: lipid nanoparticles (LNP) and adeno-associated viruses (AAV) vectors [70]. LNPs encapsulate payloads comprising a Cas effector and gRNAs delivered as DNA plasmids, RNPs, or mRNA-gRNA and support transient expression, scalable manufacturing, and repeat dosing. However, LNPs often have poor tissue targeting outside the liver, variable endosomal escape, and may even be associated with lipid-driven toxicity. AAVs facilitate more durable expression in non-dividing cells but have a size-limited capacity, can drive strong population-level immunity, and carry host-genome integration risks. For dual-use concerns, a key point is that both delivery systems can constrain permanent host genome impact; LNPs limit persistence and tissue reach, while AAVs have restricted cargo size and can be blocked by common antibodies. Efforts to expand tropism, improve escape, or increase cargo capacity for therapeutic benefit can be monitored.

### 7.2. Potential Immunogenicity of CRISPR System Components

As with all large molecule/biological modalities, as well as cell and gene therapies, there is a risk of adverse events arising from pre-existing adaptive immunity in humans—driven by prior exposure to similar bacterial proteins manifesting as anti-Cas antibodies and cytotoxic T-cells that neutralize the nuclease, eliminate Cas-expressing cells, and impair efficacy, particularly with persistent viral delivery, like AAVs [71]. Additional risks include innate inflammatory responses to bacterial genetic or protein matter, potential systemic toxicity, anaphylaxis upon redosing, and reduced therapeutic efficacy in tissues. To mitigate these risks, bioengineering strategies to reduce immunogenicity include epitope masking (e.g., R338G substitution in SaCas9 [72] or using orthologs from non-pathogenic bacteria to evade recognition). Deploying LNPs as transient non-viral delivery systems for short-lived expression, transient immunosuppression treatments, and localized administration can all help limit systemic exposure to reduce the risk of immune-related adverse event (IRAE) [73].

### 7.3. Limitations to Sample-to-Formulation

The characterization of CRISPR antivirals as enabling “sample-to-formulation” within hours to days is grounded in emerging empirical experience, although the exact timeframe depends on the platform and indication. For RNA viruses, computational gRNA design and in vitro screening can now be completed within hours of sequence availability, as demonstrated repeatedly during the SARS-CoV-2 pandemic, where Cas13 gRNA sets were designed and validated against new variants on sub-week timescales. In practice, the dominant bottlenecks are (i) gRNA synthesis and scale-up, and (ii) fit-for-purpose delivery formulation. Chemical synthesis or in vitro transcription of gRNAs (or gRNA-encoding mRNA) is typically achieved within 1–3 days in a research or rapid-response setting, after which LNP or viral vector formulation can proceed using modular, pre-optimized platforms that require only substitution of the nucleic acid payload. Formulation optimization may extend timelines when novel tissue targets, routes of administration, or safety margins are required, but this is increasingly mitigated by using standardized LNP compositions, high-throughput formulation screens, and prior Chemistry, Manufacturing, and Controls (CMC) packages that can be rapidly updated rather than built de novo. Thus, while real-world deployment still depends on manufacturing and regulatory constraints, the molecular design-to-candidate-formulation phase for CRISPR antivirals is no longer intrinsically rate-limiting and can reasonably occur on the order of hours to a few days under prepared operational conditions.

## 8. Conclusions and Outlook

The convergence of large language models for genomic and transcriptomic sequences, synthetic biology, and CRISPR antivirals is reshaping preparedness strategies for viral pathogens. As these generative AI models begin to produce functional novel viral genomes, the field is moving rapidly from recombining known motifs to exploring innovative approaches in viral construction. Given the relative ease of execution, the potential for misuse is high and requires viral defense approaches are equally agile.

Across experimental and translational studies, CRISPR antivirals have emerged as highly compelling, sequence-reprogrammable countermeasures that can be deployed at the speed of infections; Cas9 platforms can combat DNA viruses, while Cas13 and variants target RNA viruses. In vivo data across HSV-1 [24], influenza, and SARS-CoV-2 show meaningful disease suppression or viral clearance using AAV, LNP, or mRNA delivery. However, significant challenges remain, including off-target risks, vector immunogenicity, delivery constraints, incomplete viral clearance leading to persistence, or potential evolutionary escape, particularly with single gRNA delivery systems.

Nevertheless, the potential for programmable CRISPR to serve as an appropriate countermeasure is highly compelling, and a path toward feasibility can be mapped using near-term priorities:

1. Standardized CRISPR benchmarking against the most common viruses to determine adequate antiviral effectiveness and tolerability in vivo.
2. Multiplex gRNA designs to prevent viral evolutionary escape along with probabilistic escape modeling, deep multiplexing in conserved regions, and adaptive gRNA rotation coupled with surveillance screening.
3. Targeted delivery for improved tissue tropism, repeat-dose capability, and mitigation of anti-vector immunogenicity.
4. Governance and auditability with pre-cleared “safe-gRNAs” repositories, open-source safety/off-target tools, traceable design logs, regulated access to predictive models, standardized metrics for data quality, etc.
5. Regulatory pathways ready with EUA-like mechanisms for programmable endonucleases, comparability rules for iterative gRNA updates, and adaptive rapid trials.

While many unknowns remain, especially around long-term safety, host off-target or collateral activities, immunogenicity effects, and real-world escape dynamics, the direction for programmable therapies is clear: CRISPR holds significant promise as an adaptable, rapid-response foundation for antiviral preparedness. With the advent of AI-made viruses, biothreat risk is escalated, and the threat window is markedly shorter; thus the defensive timeline must be equally streamlined, scaled, and accelerated. With integrated biology, engineering, policy, and governance, CRISPR antivirals can move from laboratory modalities to a trusted public-health strategy.

It is important to mention that, while treatment modalities such as CRISPR-based antivirals programmed with target-specific guides hold promise as precise and rapid-response countermeasures against dual-use synthetic viruses engineered or AI-scripted as a biothreat, by cleaving the viral genomes directly, some major constraints would need to be addressed to make this a viable strategy in the near future. Firstly, the manufacturing of the CRISPR/Cas-gRNA payload is expensive, time-intensive, and subject to variability in the end product. As detailed below, LNP and AAV delivery approaches have limitations and risks. Rapid safety evaluation in an emergency carries the risk of missing off-target activity or immune/inflammatory reactions leading to adverse outcomes. AI-enabled virus design also remains a new development, and while the EVO system [2] did achieve functional bacteriophage genomes, modeling human infection, viral transmission, and immune evasion is still beyond current capabilities. So while the capability to create a viable, bioengineered human virus is now technically possible in principle, there remain significant gaps in expertise and execution needed to create an imminent AI-scripted biothreat.

## Figures and Tables

**Table 1:** Comparison of antiviral options to emerging viral threats.

Key Feature	CRISPR	Monoclonal Antibodies	Vaccines	Small Molecule Antivirals
Mechanism of Action	Genome cleavage to directly degrade viral genomes	Bind to viral proteins or host receptors to block entry or replication	Induce adaptive immune response against viral antigens	Inhibit viral replication enzymes or host factors
Adaptable for Unknown Viruses	Programmable across diverse pathogens with rapid gRNA reconfiguration	Predefined antigens with diverse protein modifications	Predefined viral genomes with high gene sequence variations	Target-based discovery in different biological contexts
Response Time to Outbreak	Rapid: programmable design	Moderate: requires antibody optimization	Slow: requires antigen identification and validation	Moderate: can repurpose existing drugs or screens
Sample-to-Formulation	Potentially hours to days	6–12 months	6–18 months	1–3 years
Scale-up Potential	Potentially days to weeks	Months	Months	Months
Storage Requirement	Formulation dependent ^1^	Cold chain	Cold chain	None
Manufacturing Complexity	Moderate: molecular assembly scalable	High: cell culture-based production	High: depends on platform (mRNA, vector, protein subunit)	Variable: chemical synthesis, often scalable
Breadth of Efficacy	Broadly active against multiple viral strains	Narrow, virus-specific	Variable, often strain-specific	Narrow to moderate
Safety Profile	Under development; off-target risk possible	Generally safe; infusion-related reactions possible	Established safety; varies by platform	Known pharmacology, but side effects possible
Regulatory Maturity	Experimental	Well established	Well established	Well established
Resistance Potential	Low (multi-target gRNA design possible)	Moderate (viral mutations may escape binding)	High (antigenic drift/shift)	High (point mutations in target enzymes)
Field Deployability	Potential for on-site synthesis and/or delivery	Requires cold chain and skilled personnel	Requires global manufacturing/distribution	Good for oral formulations

^1^ Storage requirements vary by formulation (e.g., lyophilized DNA-based CRISPR does not require cold chain; mRNA-based CRISPR may require −80 °C storage).

**Table 2:** Selected historical synthetically made viruses.

Virus	Genome	Year	Method	Significance	Reference
Poliovirus	(+) ssRNA	2002	Synthesis of full-length cDNA from oligos	First de novo synthesis of an infectious virus	[3]
ΦX174 Bacteriophage	ssDNA	2003	Synthesis of full-length cDNA from oligos	First de novo synthesis of bacteriophage	[4]
Influenza A (1918 strain)	(−) ssRNA	2005	Reverse genetics from historical samples	Historical re-creation; raised DURC/bioethics concerns	[6]
Horsepox virus	dsDNA	2018	Commercial DNA fragments assembled	Sparked global dual-use biosecurity debate; possible roadmap to synthetic smallpox	[5]
Bluetongue Virus	dsRNA	2008	In vitro transcribed RNA from cloned cDNAs	First dsRNA virus reconstructed via reverse genetics	[9]
Herpes Simplex Virus	dsDNA	2017	Synthetic genomics assembly	Genome-wide engineering of large DNA viruses	[10]
African swine fever virus	dsDNA	2025	BAC generation and helper virus rescue	Genome-wide engineering of large DNA virus	[11]

**Table 3:** Summary of CRISPR-based antiviral studies by viral genome.

Virus	CRISPR System	Viral Genome	Delivery Modality	References
SARS-CoV-2	Cas13	(+) sense ssRNA	mRNA, LNP (in vivo)	[13,21]
Influenza A	Cas13d	(−) sense ssRNA	mRNA, LNP (in vivo)	[13,15]
Dengue virus	Cas13b	(+) sense ssRNA	Tracking translation (in vivo)	[18]
Zika virus	Cas13b	(+) sense ssRNA	Tracking translation (in vivo)	[22]
HIV-1	Cas9	(+) sense ssRNA; proviral DNA (latent)	AAV for provirus excision	[23]
ASFV	Cas9	dsDNA	Lipid nanoparticles (LNP)	[19]
HSV-1	Cas9	dsDNA	AAV	[24,25]
HCMV	Cas9	dsDNA	AAV	[26]
HBV	Cas9	dsDNA	siP	[27]

**Table 4:** Synthetic virology and viral landmark events in DURC.

Case	Organism/ Vector	Impact Concern	Real-World Outcome	Reference
Synthetic Polio Virus	Poliovirus	Creation of synthetic virus from genetic sequence data	Demonstrated feasibility of synthesizing viruses	[3]
Mousepox with IL-4 Gene	Mousepox Virus	Increased virulence; overcame vaccine protection	Raised fears about modifying poxviruses	[53]
CRISPR Gene Drives	Mosquitoes (e.g., malaria vectors)	Potential ecosystem disruption; irreversible genetic changes	Proposals for self-regulation and moratoriums	[54]
AI-designed Novel Pathogens	Hypothetical or simulated “pathogens”	AI used to propose de novo viral blueprints	Policy discussions on dual use in AI and Biosecurity	[55]
1918 Influenza Reconstruction	Influenza A (H1N1)	Resurrecting high-virulence pandemic virus	Reconstruction raised global biosecurity and biosafety debate	[6]
H5N1 Gain of Function	Avian influenza (H5N1)	Airborne transmission and immune escape	Triggered policy moratorium and global review of GoF research	[52]
AI-designed Phages	Bacteriophage	AI-enabled synthesis of novel viral genomes	Demonstrated AI potential in synthetic biology; raised misuse concerns	[2]
Benchtop DNA Synthesis	Various	Decentralized access to build novel pathogens	Highlighted urgent need for synthetic screening policies	[56]
Horsepox Virus Synthesis	Horsepox virus (Orthopoxvirus)	Mail-ordered DNA; constructed extinct virus similar to smallpox	Ignited debate over dual-use research and synthetic-biology governance	[5]

## Data Availability

Not applicable.
